# Development and Validation of Simple RP-HPLC Method for Intracellular Determination of Fluconazole Concentration and Its Application to the Study of* Candida albicans* Azole Resistance

**DOI:** 10.1155/2015/576250

**Published:** 2015-12-09

**Authors:** Tigran K. Davtyan, Levon A. Melikyan, Nune A. Nikoyan, Hripsime P. Aleksanyan, Nairi G. Grigoryan

**Affiliations:** Scientific Center of Drug and Medical Technology Expertise JSC, Ministry of Health of Armenia, Komitas 49/4, 0051 Yerevan, Armenia

## Abstract

*Candida albicans* (strains NCTC-885-653 and ATCC-10231) long-term cultivated in the presence of antifungal agent fluconazole (FLC) and classical microbiological methods for determination of minimal inhibitory concentration (MIC) were used in this study. A simple and sensitive method based on reverse-phase high-performance liquid chromatography (RP-HPLC) has been developed for the determination of FLC intracellular concentration in* C. albicans* using tinidazole as an internal standard. Following extraction with dichloromethane, the chromatographic separation was achieved on a Machery-Nagel EC250/2 Nucleodur-100-3 C18 column by gradient elution using the mobile phase consisting of (A) 0.01 M ammonium acetate buffer, pH = 5.00, and (B) acetonitrile. Different analytical performance parameters such as linearity, precision, accuracy, limit of quantification (LOQ), and robustness were determined according to US DHHS FDA and EMEA guidelines. The method was linear for FLC (*r* = 0.9999) ranging from 100 to 10000 ng/mL. The intraday and interday precisions (relative standard deviation) were within 2.79 and 2.64%, respectively, and the accuracy (relative error) was less than 2.82%. The extraction recovery ranged from 79.3 to 85.5%. The reliable method was successfully applied to* C. albicans* azole-resistance study and it was shown that intracellular concentration of FLC correlated with a yeast drug susceptibility profile and MIC values.

## 1. Introduction

Acquired drug resistance by microorganisms poses a grave threat to human and animal health and has enormous economic consequences. Fungal pathogens, including the most common opportunistic fungal pathogen* C. albicans*, represent a particular challenge because they are eukaryotes and share many of the same mechanisms that support the growth and survival of the human host cells they infect. The number of drug classes that have unique targets in fungi is very limited, and the usefulness of current antifungal drugs is compromised by either dose-limiting host toxicity or the frequent emergence of high-grade resistance [1].

Azole compounds represent the most widely used class of antifungal drugs to treat* Candida* infections [2–4]. Azoles exert their action by inhibiting yeasts enzyme lanosterol 14*α*-demethylase and interfere with the biosynthesis of cell membrane ergosterol which causes inhibition of cell growth and finally cell death [5]. Altered interactions with the target enzyme and altered efflux pump expression are common mechanisms of azole resistance in major* Candida* species. Resistance can be mediated by increased efflux of azoles resulting from the overexpression of multiple drug resistance genes such as ATP-binding cassette transporters [6].

However, little is known about the mechanisms by which azoles and particularly FLC enter* C. albicans*. One reason for this is that the inaccessibility of the cytoplasmic face of the plasma membrane precludes direct examination of FLC intracellular transport in intact cells. To circumvent this problem, several groups have studied the ability of* C. albicans* to pump fluorescent marker compounds out of the cell. These studies have provided important insights into the energetics and kinetics of these pumps, but the fluorescent compounds used in most of these studies are unrelated structurally or functionally to the azole antifungals [7, 8]. Intracellular FLC transport is biochemically characterized by studying cellular accumulation of [^3^H]FLC [9]. The results suggest that [^3^H]FLC enters the cell by energy-independent facilitated diffusion and import levels vary among resistant clinical isolates, suggesting that import is a conserved mechanism of resistance to azole drugs in* C. albicans* [10]. Thus, determination of FLC intracellular concentration in* C. albicans* by sensitive and selective nonradioactive method is a prerequisite not only for understanding drug intracellular transport mechanisms but also for clinical monitoring of azole resistance of* C. albicans* and other medically important fungi.

A literature survey reveals some HPLC methods that are reported for the determination of FLC in pharmaceutical dosage formulations as anticipated with the variation of mobile phase, column, and detector. Different HPLC methods [11, 12] for individual assay are available for FLC in official pharmacopoeia and several LC-MS/MS methods were used for determination of FLC in human plasma [13–15]. Hence, an attempt has been made to develop a simple, efficient, and selective RP-HPLC method for intracellular determination of FLC concentration and its application to azole-resistant* C. albicans*.

## 2. Experimental

### 2.1. Chemicals and Reagents

**Figure 1:**
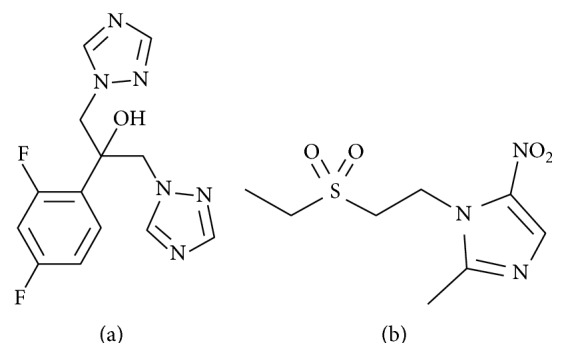
Chemical structure of fluconazole (a) and tinidazole (b).

FLC (Figure 1(a), USP RS, purity: 99.6%, batch: HОHО87), tinidazole (Figure 1(b), USP RS, purity: 99.9%, batch: 0312-QCS-12), ammonium acetate (Sigma-Aldrich, HPLC grade), sodium and potassium chloride and phosphate, millipore water, and methanol (HPLC grade, Alpha Chem Germany, purity: 99.9%), acetonitrile (HPLC grade, Alpha Chem Germany; purity: 99.9%), and sodium hydroxide, dichloromethane (Panreac, Spain, 99.9%) were used in this study.

### 2.2. Fungal Strains, Culture Media, and Antifungal Drugs

*C. albicans* NCTC-885-653 and* C*.* albicans* ATCC-10231 were purchased from the ATCC (LGC Standards-ATCC). Five different* C. albicans* clinical isolates have been obtained from Armenicum Clinical Centre (CJSC Armenicum, Yerevan, Armenia). The strains and clinical isolates were cultured in Soya-bean Casein Digest (SBD) medium (HiMedia Laboratories Ltd., India) or Sabouraud Dextrose (SD) agar (Carl Roth, Germany) with aeration at 33°С. FLC (Diflucan 2 mg/mL solution for infusion, Pfizer Inc., USA), Amphotericin B (AmB, Amphocil 100 mg, 50 mg powder for injection, Penn Pharmaceuticals Ltd., UK), and Voriconazole (VRC) substance (Liqvor Pharmaceuticals, Armenia) were used in this study. 

### 2.3. *Candida albicans *Cultivation and Biological Matrix Preparation

100 mL Erlenmeyer flasks in triplicate containing 1 × 10^5^ colony forming units (CFU) of each yeast strain cell in 100 mL SBD medium were incubated at 33°С for 48 h and yeast growth in SD agar plates at 33°C for 48 h was estimated for every 24 h by plating of 10 *μ*L and 100 *μ*L aliquots from the flasks. A total of 1 × 10^8^ CFU for each yeast strain was harvested and then cells were washed three times in ice cold PBS solution (137 mmol/L NaCl, 2.7 mmol/L KCl, 8 mmol/L Na_2_HPO_4_, and 1.46 mmol/L KH_2_PO_4_) by centrifugation at 3000 rpm at 4°C for 15 min. Aliquots of 1000 *μ*L samples were stored at −80°C and were brought to room temperature before use for method validation. Equal amounts of* C. albicans* NCTC-885-653 and ATCC-10231 strains aliquots were mixed and used for blank in biological matrix preparation. 

### 2.4. Chromatographic Conditions

Quantity analysis was acquired by using high-performance liquid chromatography Platin Blue UPLC system (Knauer, Germany) with diode array detector. Nucleodur-100-3 C18 (250 × 2 mm, 3 *μ*m packing, Machery-Nagel, Germany) column and guard column Nucleosil 120-5 C18 (CC 8/4) were employed. Gradient elution was employed using 0.01 mol/L ammonium acetate in water (pH = 5 ± 0.05, mobile phase A) and acetonitrile (mobile phase B). The flow rate was set at 0.3 mL/min and injection volume was 10 *μ*L using a full loop mode for sample injection. The temperatures of column and autosampler were maintained at 30°C and 4°C, respectively. 

### 2.5. Preparation of Standards and Quality Control Samples

Stock solutions of FLC (1 mg/mL) and tinidazole used as internal standard (IS, 1 mg/mL) were prepared independently by accurately weighing the required amounts into volumetric flasks and dissolving in methanol. The working solutions of FLC were obtained by diluting the stock solution successively with methanol. The stock solution of IS was diluted with solvent using 80 : 20 (v/v) (0.01 mol/L ammonium acetate buffer : acetonitrile) to make a working solution of 10 *μ*g/mL. All solutions were stored at 4°C and were brought to room temperature before use. For preparation of standard samples for calibration curve, 50 *μ*L of the appropriate working solutions of FLC was added to 1000 *μ*L of blank 1 × 10^8^ CFU* C. albicans* to prepare concentrations of 100, 200, 500, 1000, 2000, 5000, and 10000 ng/mL for FLC and 100 ng/mL for IS. Quality control (QC) samples at three concentration levels (low, 250 ng/mL; medium, 2500 ng/mL; high, 8000 ng/mL) were independently prepared in the same way. The standards and QC samples were freshly prepared before use.

### 2.6. Sample Preparation

A simple liquid-liquid extraction method was applied to extract the analyte and IS from* C. albicans*. Aliquots of 1000 *μ*L 1 × 10^8^ CFU* C. albicans* sample were transferred to a 10 mL polypropylene tube followed by the addition of 50 *μ*L IS working standard solution and 25 *μ*L of 6 N NaOH solution and vortex-mixed for 15 sec. 500 *μ*L of 0.01 mol/L sodium phosphate buffer (pH = 6.0) was added and vortex-mixed for 15 sec. Then, the mixture was extracted with 5 mL dichloromethane by vortex-mixing for 5 min. The supernatant was transferred to another tube after centrifugation at 3000 rpm at 20°C for 10 min and evaporated to dryness at 45°C under a gentle stream of nitrogen. Finally, the residue was reconstituted in 100 *μ*L of the solvent followed by centrifugation at 3000 rpm at 20°C for 5 min. An aliquot of 10 *μ*L of the supernatant was injected into the Platin Blue HPLC system in the full loop mode.

### 2.7. Method Validation

The method was validated for specificity, calibration curve, accuracy, precision, recovery, matrix effect, stability, and dilution effect in* C. albicans* according to the US Food and Drug Administration guidelines (US DHHS, 2001; European Medicines Agency, 2012) on bioanalytical method validation [16, 17].

#### 2.7.1. Specificity

Comparing the chromatograms of blank 1 × 10^8^ CFU* C. albicans*, 1 × 10^8^ CFU* C. albicans* sample spiked with FLC and IS, and 1 × 10^8^ CFU* C. albicans* sample after long-term cultivation of yeast cells in the presence of 1/25 MIC (20 *μ*g/mL) of FLC, no endogenous, medium components and metabolites of FLC interfered in the assay of the analyte and IS.

#### 2.7.2. Calibration Curve

The calibration curves were constructed by plotting the peak-area ratios of each analyte to IS versus biological matrix concentrations using a 1/*x* ^2^ weighted least-squares linear regression model. The acceptance criterion for each back-calculated standard concentration was ±15% deviation from the nominal value, except at the LOQ, which was within ±20%.

#### 2.7.3. Precision and Accuracy

The intraday precision and accuracy were determined by analyzing QC samples at three concentration levels (low, 250 ng/mL; medium, 2500 ng/mL; high, 8000 ng/mL) in six replicates on the same day, while the interday precision and accuracy were evaluated by analyzing QC samples at three concentration levels on three continual validation days. The precision was expressed as relative standard deviation (RSD, %) and the accuracy as the relative error (RE, %).

#### 2.7.4. Recovery and Matrix Effect

The extraction recoveries of FLC at three QC levels with six replicates were measured by comparing the peak areas from extracted samples with those from postextracted blank* C. albicans* samples spiked with the analytes at the same concentration. The extraction recovery of IS was evaluated in the same way.

The matrix effect was measured at three QC levels by comparing the peak area from the postextracted blank* C. albicans* spiked with FLC working solutions with those of corresponding standard solutions. The matrix effect of IS was evaluated using the same procedure.

#### 2.7.5. Stability

The stability of FLC in* C. albicans* was conducted at the two QC concentration levels (*n* = 5) in various storage conditions. Postpreparative stability was evaluated by analyzing the processed QC samples kept in an autosampler at 18°C for 48 h. Short-term and long-term stability were studied by analyzing QC samples exposed at room temperature for 4 h and stored at −80°C for 4 months, respectively. The freeze and thaw stability was tested by analyzing QC samples undergoing three freeze-thaw (−80°C to room temperature) cycles on three consecutive days.

#### 2.7.6. Dilution Effect and Carry-Over

Dilution effect was investigated to ensure that samples could be diluted with blank matrix without affecting the final concentration. Blank* C. albicans* samples spiked with FLC (16000 ng/mL) were diluted with pooled blank* C. albicans* at dilution factors of 2 in six replicates and analyzed. The six replicates should have precision of ≤15% and accuracy within ±15%. The carry-over was determined by injecting a blank* C. albicans* sample following the injection of an upper limit of quantification sample in three independent runs. Carry-over was considered negligible if the measured peak area was <20% of the lowest standard area.

### 2.8. Application to* Candida albicans* Azole-Resistance Study

This validated method was applied to determine the intracellular FLC concentration in* C. albicans* NCTC-885-653 and ATCC-10231 strains long-term serially cultivated in the presence of FLC. 5 × 10^4^ CFU/mL of cells in the SBD medium was incubated in separate tubes with total volume of 1.0 mL containing 1/25 of MIC concentration of FLC and incubated at 33°С for 48 h for obtaining one generation. The fungal strains were propagated in the presence or absence of selecting antifungal drugs for a total of 20 generations and MIC values for FLC, VRC, and AmB of every fifth generation of each strain were estimated.

100 mL Erlenmeyer flasks in triplicate containing 1/25 of MIC concentration of FLC and 1 × 10^5^ CFU/mL of each yeast strain (NCTC-885-653 and ATCC-10231) cell at 20th generation in 100 mL SBD medium were incubated at 33°С for 48 h and yeast growth in SD agar plates at 33°C for 48 h was estimated for every 24 h by plating of 10 *μ*L and 100 *μ*L aliquots from the flasks. A total of 1 × 10^8^ CFU yeast cells were harvested and biological matrix was prepared as described above.

For determination of the FLC intracellular concentration in* C. albicans* NCTC-885-653 and ATCC-10231 strains and 5 different* C. albicans* clinical isolates 1 × 10^6^ CFU/mL of each yeast strain in 100 mL SBD medium were incubated at 33°С for 30 min in triplicate containing 20 *μ*g/mL concentration of FLC and a total of 1 × 10^8^ CFU yeast cells were harvested for biological matrix preparation.

#### 2.8.1. Determination of Minimal Inhibitory Concentration (MIC)

Fungal strains were grown in SD agar at 33°C for 48. One colony was inoculated in 5 mL of SBD medium and washed twice with 0.9% NaCl and the fungal count was determined by spotting on SD agar. The initial concentration of the fungal suspension in the SBD medium was 5 × 10^4^ CFU/mL. 0.5 mL of suspension was inoculated into separate tubes containing serial twofold dilutions of antifungal drugs. Azole antifungal drugs (FLC and VRC) dissolved in distilled water and tested in the range of 0.95 to 1000 *μ*g/mL and AmB in the range of 0.0078 to 2 *μ*g/mL of serially diluted drugs were added to the tubes, yielding a total volume of 1 mL per tube. Drug-free medium with fungi and a fungi-free medium were used as the positive and negative controls, respectively. After incubation at 33°С for 48 h, the results were read visually, as recommended by the Clinical and Laboratory Standards Institute [18]. The MIC was considered to be the concentration that inhibited 100% of fungal growth. MIC values were confirmed by plating of 10 *μ*L and 100 *μ*L aliquots from the tubes with visual lack of growth on SD agar [19].* C. parapsilosis* ATCC-22019 were included in each susceptibility test for quality control and assessment of reproducibility testing. Each assay was performed in triplicate on three different days.

## 3. Results and Discussion

### 3.1. Method Development

The aim of this study was to develop a simple, efficient, and selective RP-HPLC method for intracellular determination of FLC concentration in* C. albicans*. Various attempts were made to separate both the analyte and IS with different pH of the mobile phase buffer and composition of methanol in the mobile phase using C-18 and C-8 stationary phase columns. To ensure great resolution between known and unknown endogenous compounds, the C-18 stationary phase with an endcapping was used. Tinidazole (Figure 1(b)) was selected as the IS since its structure, chromatographic behavior, and extraction efficiency were similar to those of the analyte. HPLC parameters, such as detection wavelength, ideal mobile phase, and their proportions and flow rate, were carefully studied. After trying different ratios of mixtures of acetonitrile and ammonium acetate buffer, the best results were achieved by using gradient elution. Both the analyte and IS displayed the best intensity and peak shape in the mobile phase containing 80 : 20 (v/v) 0.01 M ammonium acetate (solvent A) and acetonitrile (solvent B).

### 3.2. Method Validation

#### 3.2.1. Specificity and Selectivity

**Figure 2:**
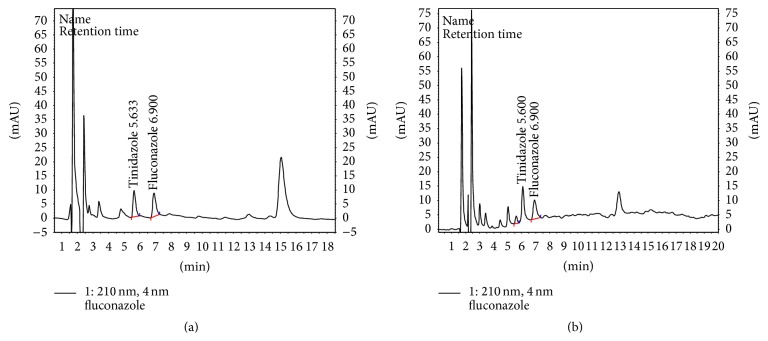
Typical chromatogram of IS and FLC (250 ng/mL) from extracted samples of blank* C. albicans* (a). Chromatographic separation of IS and FLC in* C. albicans* ATCC-10231 samples long-term cultivated in the presence of FLC.

**Figure 3:**
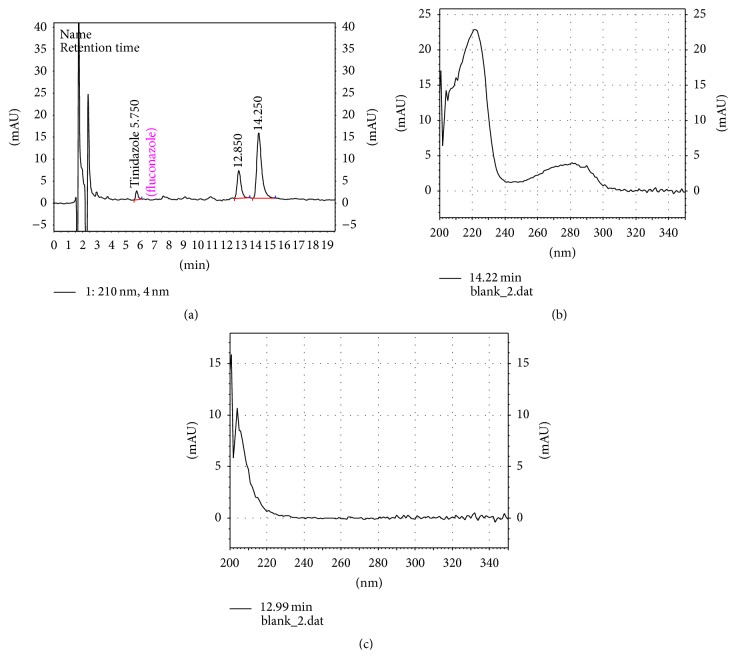
Typical chromatogram of fungi biomass blank with IS tinidazole (a). UV spectra of peak with RT at approximately 13 min (b) and UV spectra of peak with RT at approximately 15 min (c).

At a flow rate of 0.3 mL/min and the detection wavelength 210 nm, the retention time was 6.8 ± 0.02 min for FLC and 5.6 ± 0.01 min (*P* < 0.0001) for IS. The analytes peak areas were well defined and free from tailing under the described experimental conditions (Figure 2). Typical chromatograms of FLC in* C. albicans* are shown in Figure 2. Blank chromatograms with UV spectra of unidentified compounds from* C. albicans* biomass are represented in Figure 3. Obviously, there were no significant interferences from endogenous substances and metabolites of FLC at the retention time of FLC and IS.

#### 3.2.2. Calibration Curve

Calibration curve showed a satisfactory linearity in the range of 100–10000 ng/mL. A typical calibration curve equation was *y* = 0.022*x* + 3.542, with correlation coefficient of 0.9999, where *y* is the peak-area ratio of FLC to IS and *x* is the nominal concentration of FLC. The deviations of the back-calculated concentrations from their nominal values of LOQ ranged from −1.75 to 2.07% and standards other than LOQ were within −2.02 to 3.84%.

#### 3.2.3. Precision and Accuracy

The intra- and interday precision and accuracy at corresponding QC levels are summarized in Table 1. The results indicated that the method showed good precision and accuracy.

#### 3.2.4. Recovery and Matrix Effect

The extraction recoveries of FLC at the three QC levels were 79.3, 78.6, and 85.5%, respectively. The recovery of IS (100 ng/mL) was 89.4%. The extract recovery of all analytes was constant, precise, and reproducible with average percentage extraction recoveries of FLC 81 ± 4% (RSD%: 4.7). The matrix effects of FLC and IS were in the range of 85.7–90.2%, which meant that there was no significant retention time suppression or enhancement for FLC and IS.

#### 3.2.5. Stability

The stability data for FLC are presented in Table 2. The result indicated that FLC was stable under the conditions examined.

#### 3.2.6. Dilution Effect and Carry-Over

Diluted QC samples (16000 ng/mL) with six replicates were determined after dilution to the concentration of 8000 ng/mL, and the results of the tested samples were within the acceptable criteria. No carry-over was observed in the analysis of a blank plasma sample after injection the analysis of the upper calibrator (10000 ng/mL), which indicated that the carry-over effect was negligible.

### 3.3. Application to* Candida albicans* Azole-Resistance Study

This validated method was applied to determine the intracellular FLC concentration in two different* C. albicans* strains (NCTC-885-653 and ATCC-10231) long-term cultivated in the presence of FLC. The initial susceptibility profile of the selected fungal strains was investigated by determination of MIC values for antifungal drugs FLC, VRC, and AmB (Table 3). The selected* C. albicans* NCTC-885-653 strain displayed high-level azole resistance as shown by 100% fungal growth inhibition in the presence of 500 *μ*g/mL concentration of FLC and 62.5 *μ*g/mL concentration of VRC, respectively. In contrast to this strain,* C. albicans* ATCC-10231 displayed approximately 2-fold low-level azole resistance as MIC values for FLC and VRC were found to be 250 *μ*g/mL and 15.6 *μ*g/mL, respectively. However, initial susceptibility profile of the selected fungal strains to nonazole antifungal AmB was found to be identical (0.25 *μ*g/mL, Table 3). Resistance to azole drugs was experimentally induced in the selected fungal strains by long-term serial cultivation (for a total of 20 generations) of fungal cells in the presence of 1/25 of MIC concentration of FLC. The development of azole resistance was achieved for initial azole susceptible* C. albicans* ATCC-10231 strain in approximately 20 days, and the strain at 20th generation was resistant to 750 *μ*g/mL concentration of FLC and 500 *μ*g/mL concentration of VRC, respectively, and also displayed high resistance to AmB (Table 3). The susceptibility profile of the* C. albicans* NCTC-885-653 strain at 20th generation also displayed high-level azole resistance; however, MIC values for FLC and VRC were found to be 500 *μ*g/mL and 250 *μ*g/mL, respectively, and approximately 4-fold low-level AmB resistance (Table 3). 

**Figure 4:**
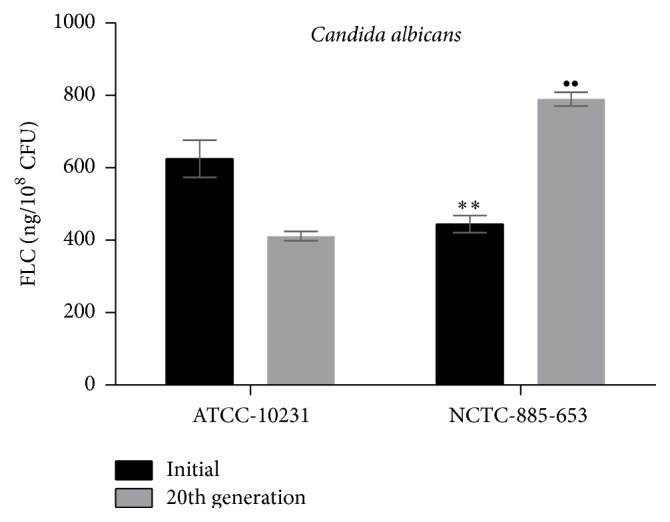
Intracellular concentrations of FLC for two different* C. albicans* strains long-term cultivated in the presence or absence of FLC. All data represent mean ± SD (error bars) for *n* = 12, for each fungal culture, and are significantly different comparing initial* Candida albicans NCTC*-*885-653* and* Candida albicans ATCC-10231* strains at ^*∗∗*^ *P* < 0.003 and comparing 20th generation of* Candida albicans NCTC-885-653* and* Candida albicans ATCC-10231* strains at ^∙∙^ *P* < 0.005, respectively.

The mean intracellular concentrations of FLC for these two* C. albicans* strains long-term cultivated in the presence or absence of FLC are shown in Figure 4. The results indicated that the intracellular concentration of FLC for initially 2-fold low-level azole-resistant* C. albicans* ATCC-10231 is statistically significantly higher comparing with the intracellular concentration of FLC for initially high-level azole-resistant* C. albicans* NCTC-885-653 (625 ± 51 ng/10^8^ CFU versus 445 ± 24 ng/10^8^ CFU, *P* < 0.003, resp.). The intracellular concentration of FLC for high-level azole-resistant* C. albicans* ATCC-10231 at 20th generation displayed 1.5-fold high resistance to antifungal action of FLC, which was found to be statistically significantly lower comparing with that of another high-level azole-resistant* C. albicans* NCTC-885-653 with 1.5-fold lower MIC value to FLC at 20th generation (412 ± 50 ng/10^8^ CFU versus 790 ± 74 ng/10^8^ CFU, *P* < 0.005, resp.). FLC uptake calculation by yeast cell indicated that single CFU of high-level azole-resistant* C. albicans* accumulated 8.1–8.7 × 10^6^ FLC molecules while low-level azole-resistant* C. albicans* accumulated 12.3–15.5 × 10^6^ FLC molecules, which is highly correlated with the 1.5–2.0-fold differences between azole susceptibility profiles of the studied fungal strains.

For future confirmation that the intracellular concentration of FLC reverse-correlated with the azole-resistance profile of fungi, we compared the MIC values and fungal FLC concentration in azole-resistant NCTC-885-653 and ATCC-10231 strains at 20th generation with five different* C. albicans* clinical isolates. For this purpose, yeast cells were propagated in the presence of 20 *μ*g/mL concentration of FLC for a total of 30 min incubation time (during which no significant difference has been observed in yeast cells survival between different strains, data not shown) and FLC intracellular concentration was determined for each strain (Table 4). The results indicated that the intracellular concentrations of FLC in azole-resistant* C. albicans* ATCC-10231 and NCTC-885-653 strains (MIC values in the 500–750 *μ*g/mL range) are statistically significantly lower, comparing with that of azole-susceptible (MIC values in the 7.8–62.5 *μ*g/mL range)* C. albicans* clinical isolates. The intracellular concentration of FLC for high-level azole-susceptible* C. albicans* clinical isolate number 1 (displayed 4-fold low resistance to FLC, comparing with isolates numbers 2–5) was found to be statistically significantly higher comparing with the rest of the azole-susceptible clinical isolates, and FLC uptake by 2-fold low resistance isolates number 2 and number 3 was statistically significantly higher, comparing with that of isolates number 4 and number 5. Thus, the obtained results clearly demonstrated that FLC uptake by yeast cell is highly correlated with the azole susceptibility profile of the studied fungal strains.

## 4. Conclusion

In the present study, a simple, sensitive, and selective RP-HPLC method for intracellular determination of fluconazole concentration in* C. albicans* was developed for the first time. A relatively simple sample preparation procedure showed greater simplicity. Baseline separation between FLC and IS ensured the accuracy of determination. The method was successfully applied to the determination of FLC intracellular concentration in different azole-resistant* C. albicans* strains for the first time and will be useful for further characterization of FLC intracellular transport mechanisms and for monitoring of drug resistance of* C. albicans* and other medically important fungi.

## Figures and Tables

**Table 1:** Intraday and interday precision and accuracy data for FLC assay in *C. albicans* matrix.

Concentration of analyte added (ng/mL)	Concentration of analyte found (ng/mL)^*∗*^	Intraday (RSD, %)^¶^	Interday (RSD, %)^¶^	Relative error (RE, %)^•^
250	252 ± 7	2.79	2.64	0.68
2500	2571 ± 61	2.35	2.99	2.82
8000	8038 ± 59	0.73	1.05	0.47

^*∗*^Mean and SD representation for *n* = 10 standard samples for each of the mentioned analytes. ^¶^RSD, % = 100 × (SD/mean). ^•^RE, % = (*E* − *T*) × (100/*T*), where *E* is the calculated concentration and *T* is the introduced concentration of the analyte.

**Table 2:** Stability of FLC assay in *C. albicans* biological matrix.

Nominal concentration (ng/mL)	Room temperature for 4 h	Stored at −80°C for 4 months	Three freeze and thaw cycles	Autosampler at 18°C for 48 h
250				
Mean ± SD	249 ± 7	254 ± 7	234 ± 4	236 ± 4
RSD, %	2.7	2.82	1.8	1.6
RE, %	−0.24	1.6	−6.32	−5.68
QC-S/QC-R, %^*∗*^	99.76	99.82	103.84	103.40
8000				
Mean ± SD	8041 ± 70	8035 ± 55	8166 ± 35.38	8162 ± 86
RSD, %	0.68	0.86	0.31	1.05
RE, %	0.44	0.51	2.08	2.02
QC-S/QC-R, %^*∗*^	99.92	99.91	102.50	100.58

Mean and SD representation for *n* = 5 standard samples for each of the mentioned analytes. ^*∗*^QC-S/QC-R, %: QC-S, samples exposed at room temperature for 4 h or stored at −80°C for 4 months or undergoing three freeze-thaw cycles or kept in an autosampler at 18°C for 48 h, and QC-R, reference samples, respectively.

**Table 3:** MIC^**∗**^ values (*μ*g/mL) for antifungal drugs FLC, VRC, and AmB of* C. albicans* initial andazole-resistantstrains, cultivated for 20 generations in the presence of FLC^¶^.

Fungal strain/antifungal drug	*C. albicans* NCTC-885-653	*C. albicans* ATCC-10231
Initial strain		
FLC	500	250
VRC	62.5	15.6
AmB	0.25	0.25
20th generation of FLC-resistant strain		
FLC	500	750
VRC	250	500
AmB	0.125	0.500

^**∗**^The MIC was considered to be the concentration of drug that inhibited 100% of fungal growth. ^¶^The fungal strains were propagated in the presence or absence of 1/25 of MIC concentration of FLC for a total of 20 generations and MIC value for FLC and VRC of each strain.

**Table 4:** MIC values for FLC and FLC intracellular concentration (*μ*g/mL) of* C. albicans* azole-resistant strains and five different *C. albicans *clinical isolates^¶^.

Fungal strains	MIC for FLC	FLC intracellular concentration
*C. albicans* clinical isolates		
Number 1	7.81	13 ± 1^•••^
Number 2	31.25	4 ± 0.2^••^
Number 3	31.25	4 ± 0.2^••^
Number 4	62.50	10 ± 1
Number 5	62.50	7 ± 0.4
*C. albicans* azole-resistant strains		
NCTC-885-653	500	0.6 ± 0.01^*∗∗∗*^
ATCC-10231	750	0.3 ± 0.02^*∗∗∗*^

^¶^ *C. albicans* azole-resistant NCTC-885-653 and ATCC-10231 strains at 20th generation and five different *C. albicans* clinical isolates strains were propagated in the presence of 20 *μ*g/mL concentration of FLC for a total of 30 min and FLC intracellular concentration was determined for each strain. The MIC was considered to be the concentration of FLC that inhibited 100% of fungal growth during 48 h incubation time. All data represent mean ± SD for *n* = 6, for each fungal culture, and are significantly different comparing *C. albicans* NCTC-885-653 and ATCC-10231 strains with clinical isolate at ^*∗∗∗*^ *P* < 0.0001 and comparing *C. albicans* clinical isolate number 1 with numbers 2–5 at ^•••^ *P* < 0.0005 and clinical isolates number 2 and number 3 with isolates number 4 and number 5 at ^••^ *P* < 0.003, respectively.
